# Leveraging interictal multimodal features and graph neural networks for automated planning of epilepsy surgery

**DOI:** 10.1093/braincomms/fcaf140

**Published:** 2025-04-16

**Authors:** Petr Nejedly, Valentina Hrtonova, Martin Pail, Jan Cimbalnik, Pavel Daniel, Vojtech Travnicek, Irena Dolezalova, Filip Mivalt, Vaclav Kremen, Pavel Jurak, Gregory A Worrell, Birgit Frauscher, Petr Klimes, Milan Brazdil

**Affiliations:** Brno Epilepsy Center, Department of Neurology, Member of ERN-EpiCARE, St. Anne’s University Hospital, Faculty of Medicine, Masaryk University, Brno 625 00, Czech Republic; Institute of Scientific Instruments, The Czech Academy of Sciences, Brno 612 00, Czech Republic; Brno Epilepsy Center, Department of Neurology, Member of ERN-EpiCARE, St. Anne’s University Hospital, Faculty of Medicine, Masaryk University, Brno 625 00, Czech Republic; Institute of Scientific Instruments, The Czech Academy of Sciences, Brno 612 00, Czech Republic; Department of Neurology, Duke University Medical Center, Durham, NC 27705, USA; Brno Epilepsy Center, Department of Neurology, Member of ERN-EpiCARE, St. Anne’s University Hospital, Faculty of Medicine, Masaryk University, Brno 625 00, Czech Republic; Institute of Scientific Instruments, The Czech Academy of Sciences, Brno 612 00, Czech Republic; International Clinical Research Center, St. Anne’s University Hospital, Brno 602 00, Czech Republic; Brno Epilepsy Center, Department of Neurology, Member of ERN-EpiCARE, St. Anne’s University Hospital, Faculty of Medicine, Masaryk University, Brno 625 00, Czech Republic; Brno Epilepsy Center, Department of Neurology, Member of ERN-EpiCARE, St. Anne’s University Hospital, Faculty of Medicine, Masaryk University, Brno 625 00, Czech Republic; Institute of Scientific Instruments, The Czech Academy of Sciences, Brno 612 00, Czech Republic; Department of Neurology, Duke University Medical Center, Durham, NC 27705, USA; Brno Epilepsy Center, Department of Neurology, Member of ERN-EpiCARE, St. Anne’s University Hospital, Faculty of Medicine, Masaryk University, Brno 625 00, Czech Republic; Department of Neurology, Mayo Clinic, Rochester, MN 55905, USA; Department of Neurology, Mayo Clinic, Rochester, MN 55905, USA; Department of Physiology and Biomedical Engineering, Mayo Clinic, Rochester, MN 55905, USA; Institute of Scientific Instruments, The Czech Academy of Sciences, Brno 612 00, Czech Republic; Department of Neurology, Mayo Clinic, Rochester, MN 55905, USA; Department of Physiology and Biomedical Engineering, Mayo Clinic, Rochester, MN 55905, USA; Department of Neurology, Duke University Medical Center, Durham, NC 27705, USA; Department of Biomedical Engineering, Pratt School of Engineering, Duke University, Durham, NC 27705, USA; Brno Epilepsy Center, Department of Neurology, Member of ERN-EpiCARE, St. Anne’s University Hospital, Faculty of Medicine, Masaryk University, Brno 625 00, Czech Republic; Institute of Scientific Instruments, The Czech Academy of Sciences, Brno 612 00, Czech Republic; Brno Epilepsy Center, Department of Neurology, Member of ERN-EpiCARE, St. Anne’s University Hospital, Faculty of Medicine, Masaryk University, Brno 625 00, Czech Republic

**Keywords:** epilepsy, surgery, graph neural networks, iEEG, MRI

## Abstract

Precise localization of the epileptogenic zone is pivotal for planning minimally invasive surgeries in drug-resistant epilepsy. Here, we present a graph neural network (GNN) framework that integrates interictal intracranial EEG features, electrode topology, and MRI features to automate epilepsy surgery planning. We retrospectively evaluated the model using leave-one-patient-out cross-validation on a dataset of 80 drug-resistant epilepsy patients treated at St. Anne's University Hospital (Brno, Czech Republic), comprising 31 patients with good postsurgical outcomes (Engel I) and 49 with poor outcomes (Engel II–IV). The GNN predictions demonstrated a significantly better (*P* < 0.05, Mann–Whitney-U test) area under the precision-recall curve in patients with good outcomes (area under the precision-recall curve: 0.69) compared with those with poor outcomes (area under the precision-recall curve: 0.33), indicating that the model captures clinically relevant targets in successful cases. In patients with poor outcomes, the graph neural network proposed alternative intervention sites that diverged from the original clinical plans, highlighting its potential to identify alternative therapeutic targets. We show that topology-aware GNNs significantly outperformed (*P* < 0.05, Wilcoxon signed-rank test) traditional neural networks while using the same intracranial EEG features, emphasizing the importance of incorporating implantation topology into predictive models.

These findings uncover the potential of GNNs to automatically suggest targets for epilepsy surgery, which can assist the clinical team during the planning process.

## Teaser

Nejedly *et al*. report a graph neural network framework for epilepsy surgery planning that integrates interictal intracranial EEG features, electrode topology, and MRI data. The model demonstrated superior predictive performance in patients with good surgical outcomes, highlighting the clinical relevance of incorporating electrode implantation topology into predictive models.

## Graphical Abstract

**Graphical Abstract:**
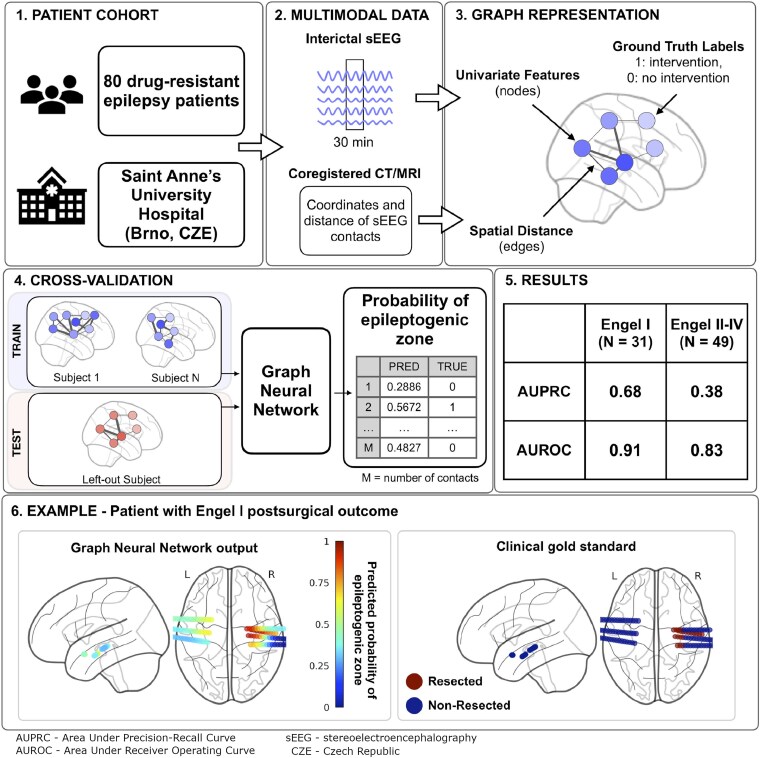

## Introduction

For patients with drug-resistant epilepsy (DRE), minimally invasive surgery is a highly specialized procedure aimed at resecting or functionally disconnecting the epileptogenic zone (EZ) from the healthy brain to prevent seizure generation. The primary goal of epilepsy surgery is to achieve seizure freedom or a significant reduction in seizure frequency and severity, thereby enhancing the patient's quality of life. The success of such procedures is heavily dependent on accurately identifying and targeting the EZ, which consists of the specific areas of the brain indispensable for generating seizures.^1^

Epilepsy surgery planning is a complex task that involves integrating various types of data, such as clinical history, MRI, scalp EEG, PET, single-photon emission computed tomography imaging, neuropsychology, and intracranial EEG (iEEG). iEEG is an indispensable tool in the pre-surgical evaluation of epilepsy patients. Unlike traditional non-invasive methods, iEEG involves the placement of electrodes directly on the brain's surface (electrocorticography) or within its tissue (stereo EEG, sEEG), providing a direct measure of neural electrical activity from specific brain regions (e.g. 150–200 recording channels) with high temporal resolution (up to 32 kHz) spanning over extended recording durations from several days to a few weeks. The primary use of iEEG is to capture ictal events and identify the seizure onset zones. Additionally, analysing interictal events, including high-frequency oscillations^2^ (HFOs), very-high-frequency oscillations,^3^ epileptiform discharges,^4^ relative entropy,^5^ and other biomarkers provides evidence for the EZ. Nevertheless, the effectiveness of various interictal iEEG biomarkers for localizing the EZ remains a subject of ongoing research.

Traditional machine learning techniques, such as logistic regression and support vector machines (SVM), based on interictal iEEG biomarkers have shown promising results in the automatic localization of the EZ.^6-11^ For instance, Cimbalnik *et al*.^6^ employed a multi-feature SVM approach to predict the seizure onset zone. At the same time, Gunnarsdottir utilized features based on functional connectivity derived from linear time-invariant systems theory and a logistic regression classifier to localize the EZ.^9^ Multimodal approaches based on MRI and high-density EEG,^12^ or MRI–PET and HFOs^13^ were also used for EZ localization. However, these methodologies lack the spatial information about iEEG implant topology that can prove critical for the accurate EZ localization. Such information can include the Montreal Neurological Institute (MNI) coordinates of electrode contacts or the distances between contacts.

In recent years, graph neural networks (GNNs) have emerged as a powerful tool for analysing complex networks represented as graphs.^14^ A graph is a mathematical object that consists of nodes and edges, where nodes represent entities and edges represent relationships between them. In EEG analysis, a graph can be constructed by representing each electrode contact as a node and the spatial distance or functional connectivity between electrodes as edges. This graph structure can capture the spatial and temporal relationships between different brain regions and the EEG signals recorded from them.

For example, applying GNNs to EEG analysis has shown promising results in various tasks, including spike detection,^15^ seizure prediction,^16^ seizure detection,^17-19^ and sleep stage classification.^20^ The potential of GNNs in epilepsy surgery planning is further enhanced by their ability to process multimodal inputs, as highlighted in a recent review by Jehi.^21^ Integrating GNNs with multimodal features presents numerous challenges and opportunities, such as advancing graph construction methods and developing more sophisticated GNN architectures.

The optimal graph construction for iEEG analysis (e.g. based on spatial or functional connectivity) and feature sets describing graph nodes (combinations of iEEG and MRI) are currently unidentified. Moreover, it remains to be seen whether incorporating spatial MNI coordinates enhances performance or introduces risks of overfitting. Including MNI coordinates could potentially bias the model towards predicting interventions in specific brain regions, such as the hippocampus, especially if the training dataset is heavily weighted with temporal epilepsy cases. Model predictions heavily influenced by MNI coordinates disregarding iEEG features would lead to undesirable clinical outcomes.

This paper introduces a multimodal GNN model based on sEEG features (power in bands, spectral features, spike rate, spike propagation, and phase-amplitude coupling) and MRI features incorporating implantation topology (Euclidean distance between electrode contacts, MNI coordinates, white matter, and grey matter distribution). The model is tested using leave-one-patient-out cross-validation on a cohort of 80 patients with DRE. The study examines how different neural network (NN) architectures (traditional NN versus GNN) and subsets of features (especially the inclusion of MNI coordinates) affect the model’s performance in EZ localization and surgery planning.

## Materials and methods

### Study design

This retrospective study presents and evaluates a new deep-learning model using a GNN approach for localizing the EZ. Our investigation aims to fulfil the following objectives:

1. Compare the performance of topology-aware GNNs model with traditional NNs
  We compare GNN, which incorporates implantation topology represented by relative distances between contacts, with a traditional feature-based NN. Both models employ identical patient data, feature extraction pipelines, and training strategies. This allows us to investigate the effect of the inclusion of implant topology on EZ localization performance.
2. Assess the impact of including MNI coordinates on model performance
  We investigate whether including spatial information in the form of MNI coordinates of electrode contacts improves model performance or introduces overfitting. We evaluate multiple configurations—with and without MNI coordinates—and compare their performance using the area under the precision-recall curve (AUPRC) and the area under the receiver operating characteristic (AUROC).
3. Evaluate clinical alignment and spatial proximity to the resected regions
  Finally, we measure how well the models’ predictions match the clinical gold standard in patient groups categorized by Engel I (good outcome) and Engel II-IV (poor outcome). As part of this evaluation, we also quantify the distance between the model's predictions for most epileptogenic electrode contacts and the surgically resected regions.

**Figure 1:**
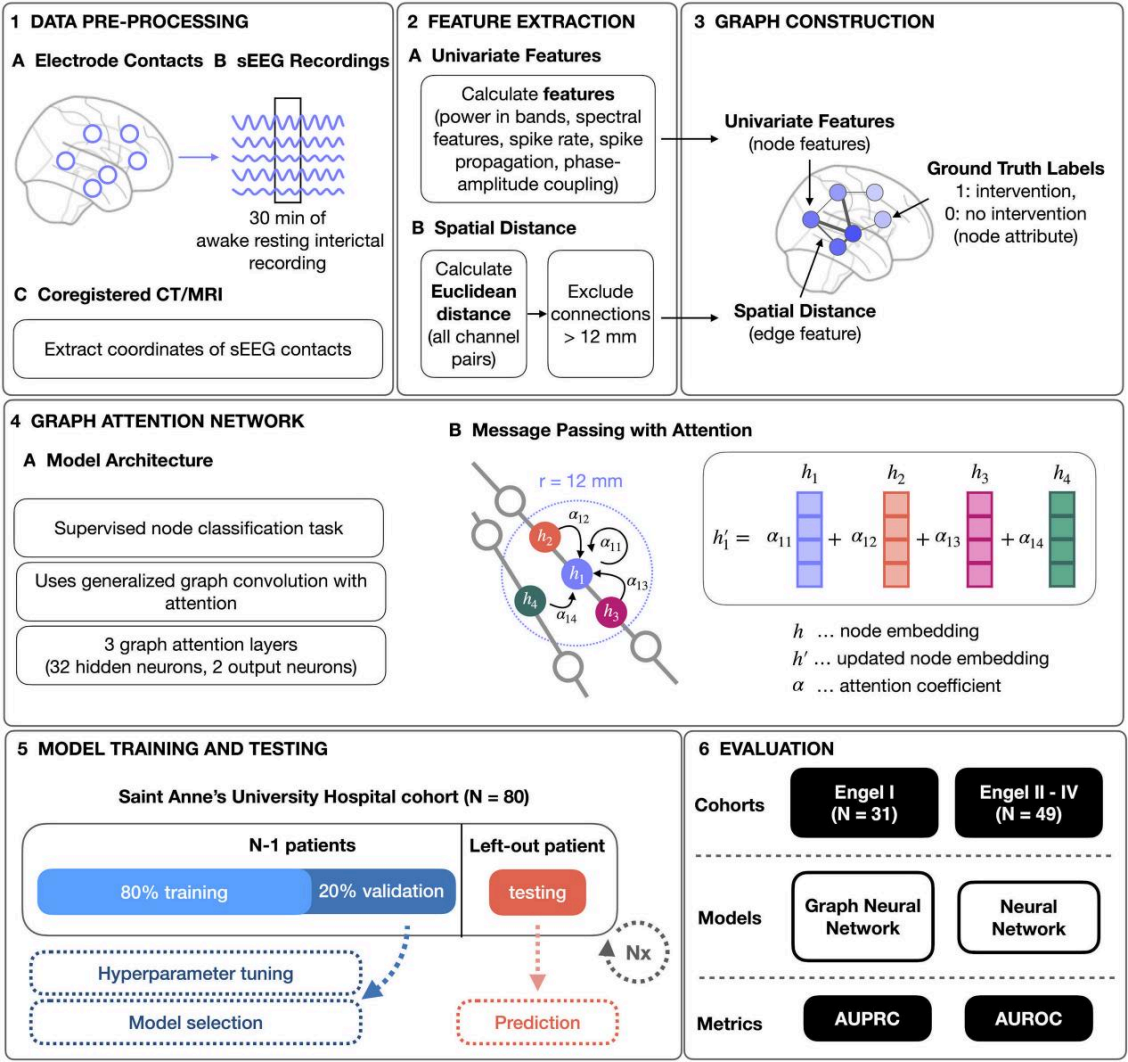
**Schematics of the study workflow.** (1) sEEG recordings are pre-processed, focusing on 30 min of awake resting interictal recording, and MNI coordinates of sEEG contacts are extracted from coregistered CT/MRI. (2) Features are extracted by calculating univariate features from the sEEG data and computing Euclidean distances between all channel pairs, excluding connections beyond 12 mm. (3) The data are structured into a graph where nodes represent the sEEG features and edges represent spatial distances between contacts. Nodes are assigned ground truth labels based on whether an intervention (resection or thermocoagulation) was performed on them or not. (4) The GNN is built from three graph attention layers that perform message passing with attention. Graph nodes representing sEEG contacts are classified as intervention versus no intervention by the network. (5) The models are trained and validated on the Saint Anne's University Hospital cohort, with 80% of data used for training and 20% for validation. A patient is left out for testing to assess the model's prediction performance. The leave one out cross validation process is repeated *N* times denoted by (Nx). (5) Comparisons are made between the GNN model and traditional NN across good (Engel I) and poor (Engel II–IV) outcome patient cohorts using the AUPRC and AUROC performance metrics.

These objectives aim to evaluate whether incorporating implant topology and MNI coordinates within GNNs can enhance the performance of EZ localization and potentially guide more effective surgical strategies in DRE. The workflow of the study is illustrated in Fig. 1.

### Dataset description

The dataset from St Anne's University Hospital, Brno, Czech Republic, was collected between 2011 and 2023 and consisted of a cohort of 124 patients. To be included in the study, patients had to satisfy the following criteria:

1. Underwent pre-surgical sEEG monitoring.
2. Received epilepsy intervention: respective surgery, radiofrequency thermocoagulation (RFTC), or both.
3. Had a preoperative MRI and a post-implantation CT/MRI for sEEG contact registration. Additionally, in cases of resection, a post-surgical MRI was required.
4. Had available follow-up (minimum 1 year) to assess whether the resection or RFTC at electrode contact sites resulted in a good outcome.^22^

The inclusion criteria were met by 80 patients (34 females, 46 males, mean age 33 ± 10 years) diagnosed with DRE indicated for epilepsy surgery (*n* = 57, 24 Engel I, 33 Engel II-IV) or RFTC (n = 10, 5 Engel I, 5 Engel II-IV), or combined RFTC and surgery (*n* = 13, 2 Engel I, 11 Engel II-IV). Epilepsy types were classified as temporal lobe (*n* = 46) or extratemporal (*n* = 34). Histopathological findings included focal cortical dysplasia in 27 patients, hippocampal sclerosis in 13 patients, unspecified classifications in 15 patients (mainly in the RFTC group), and other pathologies (e.g. gliosis, nodular heterotopia, post-traumatic, and post-meningoencephalitis changes) in the remaining patients. A comprehensive dataset description is attached in Supplementary tables.

**Figure 2:**
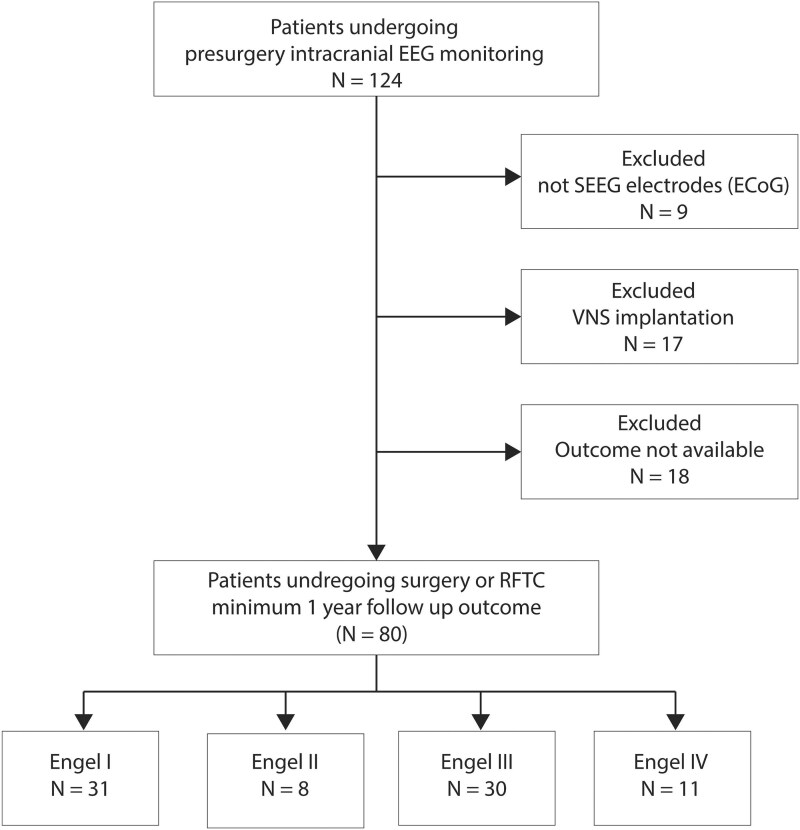
**The Preferred Reporting Items for Systematic Reviews and Meta-Analyses chart illustrates the study's patient selection process.** It details the steps from identifying potential participants (*N* = 124) to the final inclusion of patients in the analysis (*N* = 80). sEEG, stereo-EEG; ECoG, electrocorticography; VNS, vagus nerve stimulation; RFTC, radiofrequency thermocoagulation.

Patients with multifocal or bilateral lesions unsuitable for surgery were excluded due to the lack of a direct method to quantify model prediction performance numerically. The Preferred Reporting Items for Systematic Reviews and Meta-Analyses chart in Fig. 2 illustrates the patient selection process.

### Patient consent

The present study was carried out in accordance with ethical standards, and the study procedures were approved by St. Anne's University Hospital Research Ethics Committee and the Ethics Committee of Masaryk University. All subjects gave written informed consent in accordance with the Declaration of Helsinki.

### Intracranial EEG recordings

The sEEG dataset consists of 30-minute interictal sEEG recordings collected during an awake resting state according to standardized clinical protocol at St Anne's University Hospital. The recordings are typically made on the second day following the implantation of electrodes, with the day of implantation considered day zero. Partial anti-seizure medication reduction usually occurs on the evening of Day 1 or the morning of Day 2. Recordings are consistently conducted at around 10 a.m. and at least an hour away from a seizure. The acquisition system used for the measurement in the hospital was a BrainScope system (M&I, BrainScope, Czech Republic). This system allows recording up to 192 channels with a maximum 25 kHz sampling rate and common reference montage. Raw data was filtered with a 2 kHz low-pass filter and down-sampled to 5 kHz to avoid aliasing. The electrodes used in all patients from the dataset were standard intracerebral multi-contact platinum sEEG electrodes (either DIXI or ALCIS), with each patient receiving 5–16 sEEG electrodes. The location and number of electrodes were selected based on clinical reasoning. The technical parameters of the electrodes were as follows: a diameter of 0.8 mm; a contact length of 2 mm; an intercontact distance of 1.5 mm; a contact surface area of 5 mm^2^; and a number of contacts being 5, 8, 10, 12, 15, and 18. All the electrodes were MRI compatible, and their position in the brain was verified by MRI or a combination of MRI and CT examination.

### Dataset preparation

The dataset preparation involved several steps. First, a preoperative anatomical MRI was coregistered into the post-implantation CT/MRI space using SPM software^23^ and normalized to the MNI space. The superposition image was shown using MRIcron software, and MNI coordinates of sEEG contacts were manually extracted. Next, a post-resection MRI was coregistered with the preoperative MRI to delineate the resection boundaries. We also used coregistration of the post-implantation CT/MRI images with post-resection MRI to determine the resected electrode contacts. A senior clinical neurologist inspected the coregistered MRI images, and the iEEG contacts within the resected area were identified and marked. Sagging and coregistration bias were accounted for. The resected or thermocoagulated contacts were assigned to the ‘intervention’ class (Binary 1), and the untreated contacts were assigned to the ‘no intervention’ class (Binary 0). The sEEG contacts located outside of the brain were excluded from the analysis.

### Feature extraction

The node features were calculated from raw sEEG data (30-min long recordings), where the complete feature set consisted of 6 groups (described below), accounting for 31 features (exact feature description in Supplementary material). The features were selected based on a literature review of currently available features that were proven effective for the localization of the EZ. The computational library (developed by our group) for iEEG feature extraction is publicly available at (https://gitlab.com/bbeer_group/development/epycom/epycom).

Feature summary (details in Supplementary material):

1. Power in bands and spectral features: They measure the overall signal power distribution in frequency bands. Features: Total power, Spectral Centroid, Power in bands (Low Delta: 0.1–1 Hz, Delta: 1–4 Hz, Theta: 4–8 Hz, Alpha: 8–12 Hz, Beta: 12–30 Hz, Gamma: 30–80 Hz, Powerline interference: 45–65 Hz (including both 50 and 60 Hz), Ripple: 80–250 Hz, Fast ripple: 200–600 Hz), Delta–Beta ratio, Intra-patient normalized Delta power.
2. Phase amplitude coupling: This captures the relationship between the instant phase (angle of Hilbert Transform) of a low-frequency signal and the instant amplitude (amplitude of Hilbert Transform) of a high-frequency signal (phase amplitude coupling Delta: Gamma, Delta: Ripples, Delta: Fast ripples).
3. Spike rate: This includes absolute spike rates (spikes per minute) and the per-patient normalized rate on a scale (0–1). The spike detector was implemented using the methodology proposed by Janca *et al*.^4^
4. Functional connectivity metrics based on spike propagation: Metrics derived from spike propagation. The features calculated from the spike propagation matrix were based on functional connectivity measures reported by Gunnarsdottir *et al*.^9^: source, sink, centrality, hub, and authority scores^24^ characterizing the role and influence of individual contacts within the network of spike propagation.
5. MNI coordinates: Electrode contact coordinates in the standardized MNI space.
6. White and grey matter probabilities: Probabilistic measures indicate whether a contact is in white or grey matter based on WM/GM distribution atlas based on MNI coordinates.

The sEEG features were chosen to capture the local and global structure of sEEG activities. Therefore, some features are normalized per patient, while others are expressed in absolute values. For example, signal power is normalized per patient, with 0.0 assigned to the contact with the lowest signal power and 1.0 to the contact with the highest signal power.

HFOs, a common interictal biomarker, were not included as a feature of the model as recent studies^8,25^ have demonstrated that HFOs do not provide additional information for localization compared with interictal spikes. However, localization results based solely on the HFO rate detected by the RMS detector^26,27^ are provided for comparison.

### Graph construction

The graph was generated using sEEG features and the spatial distances between sEEG contacts. Node attributes were defined by the sEEG features, while edges represented the Euclidean distances between contacts. To determine the neighbourhood of each sEEG contact, we applied a heuristic threshold, considering any edge within 12 mm (including those connecting contacts from different electrodes) as part of the neighbourhood. The distance was determined using a centre-to-centre intercontact step of 3.5 mm, with the understanding that the sEEG signal is primarily influenced by its surroundings (approximately distance of ±3 contacts, equating to 10.5 mm). To accommodate potential MRI/CT coregistration errors, this value was adjusted to 12 mm. This value is also supported by observations that sEEG is sensitive to neural activities approximately up to a distance of 10 mm.^28^ The ‘Discussion’ section further describes the effects of distance on model performance.

### Model architecture

A simple GNN architecture utilizing generalized graph convolution with attention^29^ was employed as the classification model. The model consisted of three layers with 32 hidden neurones and two output neurones since each node is classified into one of two classes (intervention/no intervention). The network architecture was purposely set as very simple to prevent overfitting since the training dataset is small compared with datasets used in other scientific fields.

### The dataset split into training, validation, and testing

In line with best practices in machine learning, a leave-one-patient-out cross-validation approach was employed. This method ensures that data from the test patient does not influence the model's training and validation phases, demonstrating the model's generalizability and providing unbiased results when applied to novel patients.

The rest of the patients’ data (*N*−1) were split into 5-folds, each containing 20% of the patients. Four folds were used for training, while 1-fold served as the validation set to detect overfitting and facilitate early stopping. This procedure was rotated five times, each time producing a new model. Together, these five models form a classification ensemble, effectively maximizing the information capacity of the dataset.

### Model training and validation

The model was trained over 30 epochs using the Cross-Entropy Loss with rescaled weights (1:10 weights assigned to negative:positive class) and the Adam optimizer, with a learning rate set at 0.005 and an L2 regularization weight decay of 0.0001. After each training epoch, the validation loss and AUPRC were monitored, an early stopping strategy was used to select the model with the best validation score.

### Model testing, evaluation, and statistical analysis

**Figure 3:**
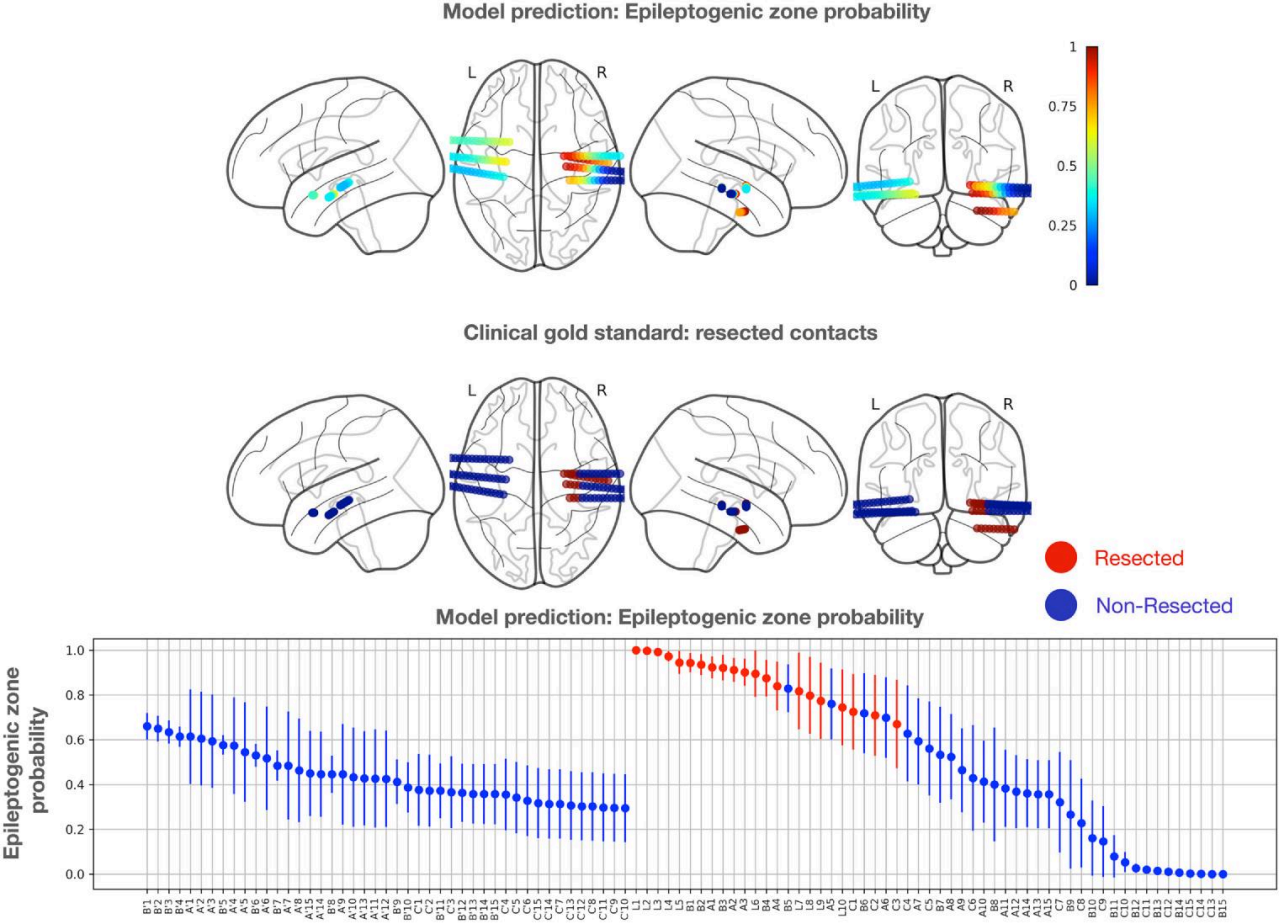
**A case study demonstrating retrospective model predictions for a patient with an Engel I outcome.** The top graph uses a colour gradient to represent the predicted probability of the epileptogenic zone (EZ), where a value of 1 signifies a recommended intervention site, and 0 indicates healthy tissue with no need for an intervention. The middle graph shows the gold standard, with red contacts representing resected areas and blue contacts indicating non-resected areas. In the bottom graph, the *x*-axis displays individual stereo-EEG electrode contacts, ordered by their average probability as predicted by the classification ensemble. Error bars represent the standard deviation of probabilities across ensemble models. Red-marked electrode contacts indicate surgically resected areas. The graph is divided into two sections, corresponding to contacts from the left and right hemispheres.

In the study, the output of the classification ensemble was derived from the average outputs of five cross-validation models to predict intervention electrode contacts for a novel patient (leave-one-patient-out testing) completely excluded from training and validation. An example of model output and clinical gold standard is depicted in Fig. 3. The model output probabilities were compared with the clinical gold standard and quantified by reporting the AUPRC and AUROC to provide objective and comprehensive evaluation based on methodology from Hrtonova *et al*.^30^

Given the typical imbalance in EZ localization datasets, where ∼90% of electrode contacts are healthy and only 10% are epileptogenic, AUROC scores can be misleading as they are heavily influenced by the majority class of non-epileptogenic contacts. That is why we emphasize the importance of the AUPRC, a metric that depends on sensitivity (SEN) and positive predictive value (PPV), providing a more focused evaluation of the model's performance. To ensure a comprehensive evaluation of the model's effectiveness in handling imbalanced datasets, the AUPRC and AUROC of a random chance classifier, which are essential to provide a direct comparison and context were reported. Chance-level performance in a binary classification task is an AUROC of 0.5, and the AUPRC is equal to the proportion of positive samples in the dataset.

In the statistical analysis, we utilized the Wilcoxon signed-rank test to determine if there is a statistically significant difference in performance based on AUPRC between the models tested on Engel I patients. The significant differences are accompanied by effect size represented by Cohen's *d*-value, for which the effect can be interpreted as negligible (*d* < 0.15), small (0.15 ≤ *d* < 0.33), medium (0.33 ≤ *d* < 0.47), or large (*d* ≥ 0.47).^31^ Additionally, we assess whether there is a significant performance difference between Engel I patients and those in Engel II–IV groups with the Mann–Whitney U-test.

### Discretization of model outputs

A two-step heuristic approach was implemented to convert continuous model output probabilities into binary decisions recommending intervention. The discretization process was defined as follows:

1. Probability thresholding: Initially, electrode contacts are identified where the model's output probability surpasses a predefined global threshold.
2. Spatial neighbourhood selection: For each contact identified in the first step, all surrounding contacts within a predefined resection radius are also selected for potential intervention.

The intervention suggestions were compared with the clinical gold standard using two key metrics. SEN that measures the proportion of actual interventions correctly suggested by the model and PPV, which assesses the proportion of intervention suggestions that were correct.

### Evaluating spatial alignment of the predicted epileptogenic zone and intervention area

Establishing the precise spatial correspondence between the model's predicted EZ and the intervened region is often challenging, particularly due to the probabilistic nature of the model's output and the discretized representation of electrode contacts in MNI space. Therefore, in this study, we focus on the single electrode contact identified by the model as having the highest epileptogenic probability and determine its spatial distance to the nearest surgically resected contact. Let *P*_max​ denote the MNI coordinates (*x*,*y*,*z*) of the most epileptogenic contact, and let E_i represent the coordinates of the *i*-th resected contact. The Euclidean distance is then calculated as:


$$\mathrm{distance}=\mathrm{mi}n_{i\;\in \{\mathrm{resected}\}}\|P_{\max}-E_{i}{\|}_{2}$$

In cases where the electrode contact with the highest epileptogenic probability falls within the intervention zone, the distance is zero. Otherwise, the distance corresponds to the Euclidean distance (in millimetres) between that contact and the nearest intervention contact. It is important to note that this single-contact metric may be unsuitable for patients with multifocal or bilateral epileptogenic foci (example in discussion), given that it only considers the contact with the highest predicted EZ probability.

## Results

In this study, we assessed the effectiveness of GNNs in automating epilepsy surgery planning by integrating spatial features, MRI data, and sEEG biomarkers. To enable direct comparison with state-of-the-art methods, we benchmarked our models against spike rate (Janca detector) and HFO rate (RMS detector), using identical datasets (Table 1). Statistical tests evaluating differences in AUPRC performance across the proposed models are presented in Table 2.

**Table 1:** GNN outperformed traditional NNs and baseline models in localizing intervention (resected or thermocoagulated) electrode contacts

		AUPRC		AUROC	
Model ID	Model description	ENGEL I (*n* = 31)	ENGEL II–IV (*n* = 49)	ENGEL I (*n* = 31)	ENGEL II–IV (*n* = 49)
	Random chance classifier	0.0965	0.0761	0.500	0.500
	Spike rate (Janca detector)	0.4753	0.2623	0.8250	0.7859
	HFO rate (RMS detector)	0.3797	0.2213	0.7732	0.7568
1	GNN (all features + MNI + distance)	0.6897	0.3845	0.9105	0.8387
2	GNN (all features + distance)	0.6017	0.3861	0.8317	0.7710
3	NN (all features + MNI)	0.6383	0.3133	0.9044	0.7940
4	NN (all features)	0.4367	0.2976	0.8301	0.7783
5	GNN (spike rate only + MNI + distance)	0.6419	0.2775	0.9302	0.8506
6	GNN (spike rate only + distance)	0.5111	0.2589	0.8636	0.8107
7	NN (spike rate only + MNI)	0.5013	0.2327	0.8939	0.8002
8	NN (spike rate only)	0.4753	0.2623	0.8250	0.7859

Abbreviations: AUPRC, area under the precision-recall curve; AUROC, area under the receiver operating characteristic curve; GNN, graph neural network; NN, neural network; MNI, Montreal Neurological Institute coordinates.

**Table 2:** *P*-value comparison of AUPRC scores in Engel I patients across models using the Wilcoxon signed-rank test

Model	ID	1	2	3	4	5	6	7	8
GNN (all features + MNI + distance)	1		0.098	0.247	* 0.010 *d* = 0.54	0.489	0.253	0.298	* 0.018 d = 0.50
GNN (all features + distance)	2			0.900	* 0.023 *d* = 0.48	0.797	0.688	0.779	0.063
NN (all features + MNI)	3				0.130	0.900	1.000	0.410	0.299
NN (all features)	4					0.163	0.135	0.568	0.309
GNN (spike rate only + MNI + distance)	5						0.779	0.405	0.388
GNN (spike rate only + distance)	6							0.721	0.120
NN (spike rate only + MNI)	7								0.650
NN (spike rate only)	8								

*P*-values for statistical comparisons of AUPRC scores across all models from Table 1 are presented, calculated using the Wilcoxon signed-rank test. Statistically significant differences (*P* < 0.05) are marked with *, and Cohen's *d*-value represents effect sizes. AUPRC, area under the precision-recall curve; GNN, graph neural network; NN, neural network; MNI, Montreal Neurological Institute coordinates.

The numerical results (Table 1) for AUROC and AUPRC provide insights into the influence of individual features on neural network performance, feature selection, and the effects of MNI coordinates (Table 3). These findings are elaborated upon in the subsequent discussion.

**Table 3:** Models trained exclusively on MNI coordinates achieved better-than-random performance, indicating overtraining towards predominantly resected regions

	AUPRC		AUROC	
Model description	ENGEL I (*n* = 31)	ENGEL II–IV (*n* = 49)	ENGEL I (*n* = 31)	ENGEL II–IV (*n* = 49)
Random chance classifier	0.0965	0.0761	0.5	0.5
GNN (MNI + distance)	0.2875	0.1259	0.7571	0.6318
NN (MNI)	0.2732	0.1573	0.8276	0.7227

Performance metrics are shown for GNN and traditional NN models predicting intervention targets using MNI coordinates. AUPRC, area under the precision-recall curve; AUROC, area under the receiver operating characteristic curve; GNN, graph neural network; NN, neural network; MNI, Montreal Neurological Institute coordinates.

### Best performing model

We found that the GNN model (Table 1, Model 1), which was trained on all available sEEG features and MNI coordinates, achieved the highest AUPRC (0.6897) among all tested configurations. Notably, its AUPRC improvement was statistically significant (Table 2, Model 1 versus 4) compared with the state-of-the-art NN model (Table 1, Model 4), which scored an AUPRC of 0.4367. Similarly, the GNN model also showed a statistically significant performance gain over spike rate—the best-performing single feature (Table 2, Model 1 versus 8)—which achieved an AUPRC of 0.4753.

To explain the sources of the observed AUPRC performance gains of model 1—specifically, whether they arise from differences in model architecture (GNN versus NN) or from incorporating MNI coordinates—we conducted a series of additional experiments using multiple model configurations (Models 2–4).

To evaluate the impact of spike rate on model performance, we tested additional model configurations (Models 5–8), revealing that Model 5 (reduced sEEG feature set) achieved AUPRC performance comparable to the best-performing Model 1. This confirms that spike rate is the primary driver for model decisions, while other sEEG features play a supportive role in the model's decision process.

### Effect of neural network architecture (graph neural network versus neural network)

To examine the effects on GNN architecture, we trained the GNN model without MNI coordinates (Table 1, Model 2) and compared its performance to the state-of-the-art NN approach (Table 1, Model 4). We observed a statistically significant improvement in AUPRC with a large effect size (Table 2, model 2 versus 4), indicating that incorporating inter-contact distances enhances predictive performance.

### Effect of Montreal Neurological Institute coordinates

To examine the effects of MNI coordinate inclusion, we trained the NN model with MNI coordinates (Table 1, Model 3) and compared its performance to the state-of-the-art NN approach (Model 4). Although the AUPRC for Model 3 increased by 0.2 (from 0.4367 to 0.6383), this improvement did not reach statistical significance (Table 2, Model 3 versus 4).

Next, we examined whether models could be overtrained to predict interventions in specific brain regions by training a model exclusively on MNI coordinates. While such an approach has limited practical utility, it effectively illustrates how datasets with a large number of temporal lobe epilepsy (TLE) patients can bias models towards temporal lobe structures, such as the hippocampus. Notably, even this coordinate-only model achieved AUPRC and AUROC scores above random chance (Table 3). Indicating the capability of models to overtrain towards specific MNI coordinates.

### Output binarization

To translate probabilistic predictions of EZ into actionable clinical decisions, we applied thresholding to the output probabilities. This approach binarized the model outputs, assigning a binary label to each contact, indicating whether it suggests intervention. The binary decisions were evaluated by calculating SEN and PPV across various probability thresholds and resection radii (defined as the spatial neighbourhood of contacts exceeding the threshold).

Table 4 summarizes the sensitivity (SEN), positive predictive value (PPV), and specificity (SPC) of the best-performing model (GNN Model 1 from Table 1) for Engel I patients. These metrics were analysed across a range of probability thresholds (0.75–0.90) and resection radii (3–12 mm). This analysis highlights the trade-off between SEN and precision in intervention planning. For instance, using a probability threshold of 0.8 with a radius of 9 mm resulted in a SEN of 66%, SPC of 91% and a PPV of 44%.

**Table 4:** Group statistics showing median of SEN, SPC, and PPV of the best-performing model (GNN Model 1, Table 1) in ENGEL I patients across varying probability thresholds and resection radii

SEN/SPC/PPV (%)		Probability threshold			
		0.75	0.8	0.85	0.90
Resection radius (mm)	3	66/92/44	57/93/51	35/95/51	28/97/64
	6	69/91/44	64/93/44	42/94/46	29/96/55
	9	70/89/41	66/91/44	48/93/42	33/95/51
	12	71/88/37	68/90/36	50/92/42	38/93/46

Probabilistic predictions of epileptogenicity were converted into binary decisions through thresholding, with intervention suggested for contacts above the threshold. The table presents SEN and PPV as functions of the probability threshold (0.75–0.90) and the resection radius (3–12 mm). SEN, Sensitivity; SPC, Specificity; PPV, Positive Predictive Value; GNN, Graph neural network.

### Alignment between predicted epileptogenic zone and intervention area

**Figure 4:**
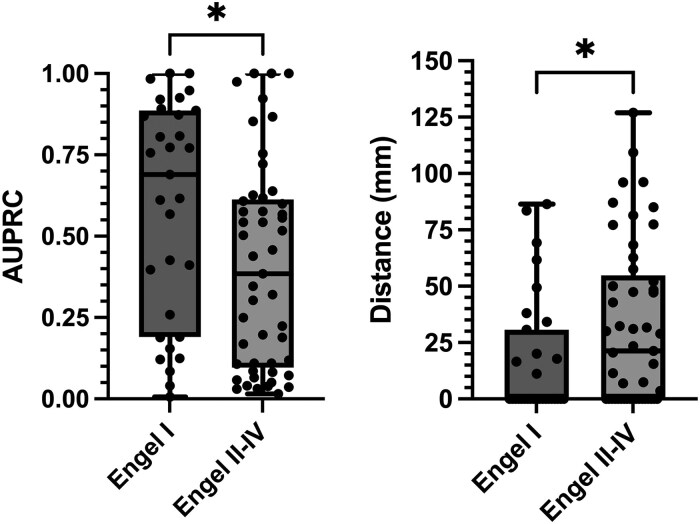
**Performance metrics for best performing model stratified by Engel outcomes.** The left boxplot illustrates the distribution of AUPRC values for the Engel I (*N* = 31) and Engel II–IV (*N* = 49) groups, with each dot representing an individual patient. The Engel I group shows significantly higher AUPRC values compared with the Engel II–IV group (*P* = 0.015; Mann–Whitney U-test), indicating a better alignment between model predictions and clinical decisions (gold standard). The right boxplot depicts the distances (in mm) between the contact with the highest predicted epileptogenic probability (*P*_max) and the nearest contact targeted for clinician intervention. The Engel I group has significantly lower distances compared with the Engel II–IV group (*P* = 0.03; Mann–Whitney U-test), suggesting a closer match between model predictions and actual intervention sites. EZ, Epileptogenic Zone, *P*_max, Maximum probability of epileptogenicity, GNN, graph neural network, MNI, Montreal Neurological Institute coordinate, AUPRC, area under the precision-recall curve.

Figure 4 shows the distribution of AUPRC values and spatial distances between the contact with the highest model-predicted EZ probability (*P*_max) and the nearest intervention contact, based on the best-performing model (GNN Model 1, Table 1). The Engel I group has significantly higher AUPRC values, indicating better predictive performance compared with the Engel II-IV group (*P* = 0.015; Mann–Whitney U-test). For Engel I patients, the median distance is zero, meaning that in most cases, the *P*_max contact is within the intervention zone. In contrast, Engel II–IV patients have a distance between *P*_max and resection approximately 20 mm (median), suggesting less accurate targeting of the intervention zone. The differences in distances are statistically significant (*P* = 0.03; Mann–Whitney U-test). Both groups include some patients with distances greater than 40 mm, which often occurs when the predicted targets are on the opposite side of the brain, particularly in cases of suspected bitemporal epilepsy. This phenomenon is further explored in the ‘Discussion’ section.

## Discussion

We proposed an application of GNNs for automating epilepsy intervention planning by leveraging sEEG features, MNI coordinates of the sEEG contacts, and spatial distances between the sEEG contacts derived from MRI data. The effectiveness of GNNs in localizing the EZ (defined as sEEG contacts that were resected or thermocoagulated in a clinical intervention) was retrospectively tested on a dataset of 80 patients who underwent epilepsy surgery and the proposed approach was compared with traditional NNs (Table 1) and state-of-the-art methods (Table 5). Our findings highlight the advantages of GNNs, particularly their ability to account for the patient-specific sEEG implantation topology in the analysis. This approach effectively allows the model to account for the heterogeneity of implantations, identified as one of the main challenges in deploying quantitative methods in epilepsy surgery in a review by Bernebei *et al*.^32^

**Table 5:** Comparison of state-of-the-art methods for localizing the epileptogenic zone from intracranial EEG across partially overlapping and distinct datasets

Method	Outcome	N. patients	AUPRC	AUPRC (random chance)	AUROC
Studies on partially overlapping datasets					
GNN (this paper)	Good	31	0.68	0.0965	0.91
	Poor	49	0.38	0.0761	0.83
SVM (Cimbalnik at al.)^6^	Good	9			0.842
	Poor	7			0.776
SVM (Chybowski *et al*.)^7^	Good	25	0.49	0.062	
	Poor	Not included			
LR (Klimes *et al*.)^8^	Good	18	0.608	0.098	0.827
	Poor	32			
Relative Entropy (Travnicek *et al*.)^5^	Good	39	0.43		0.85
	Poor	Not included			
Studies on other datasets					
LR (Gunnarsdottir *et al*.)^9^	Good	28			0.77
	Poor	37			
Classification Trees (Bernabei *et al*.)^10^	Good	38	0.25		
	Poor	22	0.14		
	All	60			0.77
LR (Conrad *et al*.)^11^	Good	24			0.82
	Poor	15			
	All	44			0.83

The proposed GNN method demonstrates the highest metric scores, achieving an AUPRC of 0.68 and AUROC of 0.91 for “Good” outcomes outperforming other approaches such as SVM, LR, and Classification Trees. AUPRC, area under the precision-recall curve; AUROC, area under the receiver operating characteristic curve; GNN, graph neural network; SVM, support vector machine; LR, logistic regression.

In this study, we showed that:

- GNNs provide a robust and flexible framework for integrating multimodal features. This includes combining univariate and bivariate sEEG features with intercontact distances and other diverse data types.
- GNNs consistently outperformed standard NNs (measured by AUPRC and AUROC, Table 1). Additionally, they surpassed other state-of-the-art approaches for automatic EZ localization (Table 5), establishing their effectiveness for automatic epilepsy surgery planning.
- Incorporating MNI coordinates of sEEG contacts into the feature set boosts performance. However, it may lead to overfitting specific brain structures, as shown in Table 3.
- Including patients with both good and poor postsurgical outcomes in model training improved the model's level of generalizability, highlighting the importance of diverse training data.

### State-of-the-art comparison

Comparing artificial intelligence methods across different datasets is generally challenging due to inherent dataset heterogeneity. For instance, variations in the number of implanted sEEG contacts and the number of contacts resected significantly impact the random chance levels for the AUPRC. Additionally, the inclusion criteria for the patient cohort, such as the ratio of temporal to extratemporal cases, significantly influences the reported performance.

Table 2 provides directly comparable results for EZ localization based on automated detection of spike rate (Janca detector) and HFO rate (RMS detector). The performance of the HFO detector in this study is numerically comparable to the findings reported by Gunnarsdottir *et al*., which documented an AUROC of 0.71 ± 0.10.

Table 5 presents a comparison of our results with similar methodologies previously published both within and outside our research group. However, direct comparisons are challenging due to differences in dataset sizes, institutional biases, and varying ratios of temporal to extratemporal cases across studies.

### Impact of patient selection on model performance

**Figure 5:**
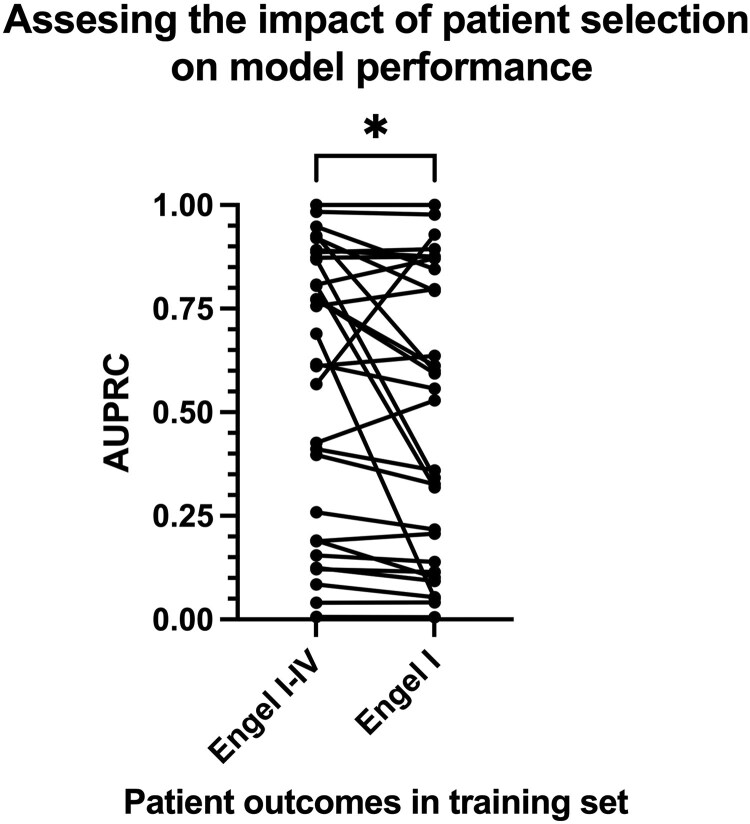
**Impact of training data selection on model performance.** Comparison of the best-performing GNN model trained on patients across all outcome groups (Engel I–V, *N* = 80) versus a model trained exclusively on good outcome patients (Engel I, *N* = 31). Each data point represents the AUPRC for an individual Engel I outcome patient in a leave-one-out cross-validation experiment. The left side illustrates AUPRC values when the model is trained using both good (Engel I) and poor (Engel II–IV) outcome patients, while the right side shows AUPRC when trained solely on good outcome patients. Lines connect predictions for the same patient across both models, emphasizing the within-subject comparison. A statistically significant difference in performance is observed, favouring the model trained on all outcomes (Wilcoxon signed-rank test, *P* = 0.014, Cohen's *d* = 0.39).

Although including patients with both good and poor outcomes in model training remains controversial due to concerns about clinical data reliability, the surgical resections in these cases were guided by key clinical indicators of EZs, such as interictal spikes, focal slowing, and in particular seizure onset areas. Our preliminary findings suggest that incorporating data from patients with good and poor outcomes can enhance model performance relative to training solely on patients with good outcomes. The Wilcoxon signed-rank test was used to test for AUPRC differences in Engel I groups, indicating a statistically significant difference (*P* = 0.011, Cohen's *d* = 0.42). Since surgeries for poor-outcome patients are not undertaken randomly, we believe these datasets also contain valuable information for model training. Consequently, excluding patients with poor outcomes substantially reduces the dataset size, which may compromise the model's generalizability (Fig. 5). However, these conclusions should be viewed as a hypothesis that requires further investigation and validation on larger datasets.

### Graph construction, model architecture

In this paper, we used a GNN based on the graph attention layer,^29^ where the adjacency matrix is derived from the spatial distances between contacts (a neighbourhood of 12 mm). Experimentally, we found that increasing the threshold for spatial distance did not improve results. In general, we recommend using less connected graphs since GNNs have an over-smoothing problem,^33^ which refers to the phenomenon that increasing node connections or model depth leads to the averaging of the information, leading to decreasing performances.

We would like to highlight the need to investigate other GNN architectures, e.g. graph convolutional networks^34^ or point cloud transformer architectures,^35^ that would also allow for integrating electrode topology and MNI coordinates into classification models. Additionally, it is essential to explore whether other adjacency metrics, such as those based on functional metrics, could yield more effective results.

### Effect of Montreal Neurological Institute coordinates

In Table 3, we showed that training models solely on MNI coordinates of sEEG contacts achieved superior performance compared with random chance classifiers. These models often identified the hippocampal area as a key region for resection in temporal epilepsy patients and predicted contacts in this region as likely intervention targets. When these models were evaluated in TLE DRE patients, they performed exceptionally well. This suggests that including MNI coordinates might lead to model overtraining, which could result in recommending interventions in brain regions that are commonly targeted. This is an undesirable outcome in practice because the models might predict interventions based on the most frequently intervened structures in the training patient cohorts.

Employing the GNN without MNI coordinates (Model 2) instead of the full model (Model 1), particularly in smaller datasets where overfitting poses a significant challenge, effectively minimizes the clinically unwanted scenario of predicting intervention based on most frequently intervened structures in the training patient cohorts. At the same time, we believe that incorporating MNI coordinates may offer significant advantages in large datasets, where the risk of overtraining is likely to be less significant. Ultimately, the inclusion of MNI coordinates has the potential to enable models to train a ‘spatial dependent biomarker atlas’ facilitating the differentiation of biomarkers as normal in some regions while pathological in others. This study serves as a pilot effort, emphasizing the need for further investigation using large, multicentric datasets.

### Strengths and limitations

This study used a relatively large dataset compared with actual state-of-the-art reports in sEEG studies (Table 5). Although precautions were taken to prevent overfitting (leave-one-out patient cross-validation and ensemble forming), the dataset from a single centre still poses a risk to institution-specific biases.

From the sEEG monitoring point of view, we utilized only 30-minute-long recordings collected during the awake resting state. The evaluated features were aggregated over the entire recording period. Future studies should explore the temporal variability of these features over more extended periods and across multiple behavioural states (e.g. non-REM sleep). As was recently shown, the vigilance state affects EZ localization results.^7,36^

**Figure 6:**
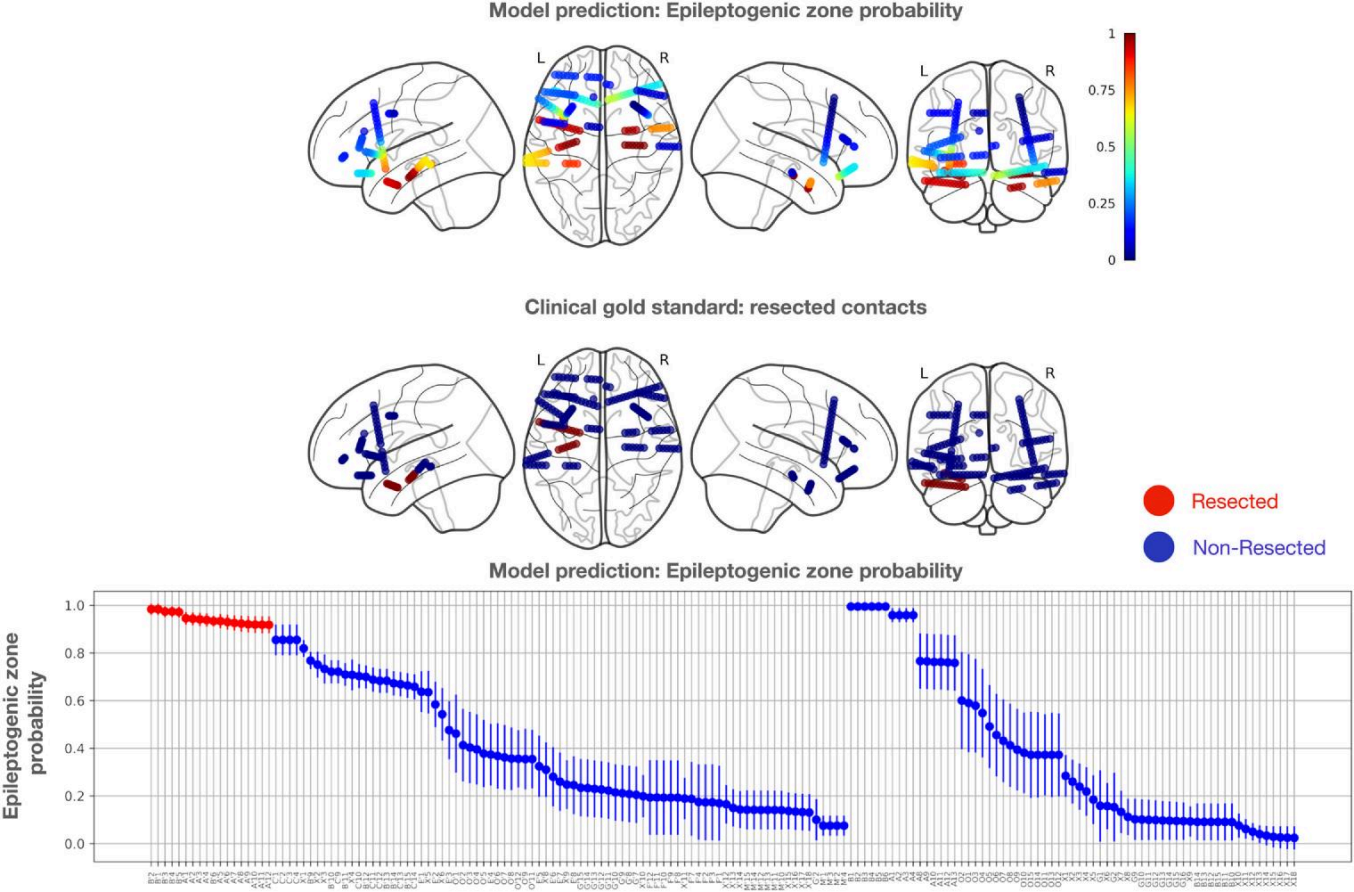
**A case study illustrating challenges in processing data from patients with suspected multifocal or bitemporal epilepsy.** The model's predictions are shown for a patient who underwent surgery with an Engel I outcome. MRI findings suggested left hippocampal sclerosis, and clinical interpretation of sEEG monitoring identified the seizure onset zone in the left hippocampus, with interictal epileptiform discharges observed bilaterally. Despite this, the model, which processes only interictal data, predicted bilaterally elevated probabilities in the temporal region. Performance metrics for the model included an AUPRC of 0.45 and AUROC of 0.90. AUPRC, area under the precision-recall curve; AUROC, area under the receiver operating characteristic curve.

The methodology faces challenges in processing data from patients with suspected multifocal or bitemporal epilepsy. For example, Fig. 6 highlights the model's predictions for a patient who achieved an Engel I outcome following surgery. MRI findings indicated left hippocampal sclerosis and sEEG monitoring clinically identified the seizure onset zone within the left hippocampus despite the presence of bilateral interictal epileptiform discharges. However, the model, which exclusively utilizes interictal data, predicted elevated probabilities bilaterally in the temporal region. The performance metrics for this case revealed an AUROC of 0.90 and an AUPRC of 0.45. This discrepancy can be largely attributed to the model's lack of access to comprehensive clinical information. These findings emphasize the need for next-generation models to incorporate additional data, such as seizure onset zones and MRI findings, to improve accuracy and reliability.

## Conclusion

The application of GNNs in epilepsy surgery planning shows significant promise, particularly in enhancing the data and information that the team of physicians can use to improve precision and the outcomes of surgical interventions. GNNs allow for integrating the patient-specific implantation topology with sEEG and MRI features. This integration represents a valuable advancement over current state-of-the-art methods, offering a pathway to more effective and precise surgical intervention planning. Multicenter studies should be conducted to assess the robustness and generalizability of GNN-based approaches to validate the benefits of GNNs.

## Supplementary Material

fcaf140_Supplementary_Data

## Data Availability

The overall size of the used data exceeds several hundred gigabytes and cannot be publicly shared. However, data might be obtained upon reasonable request by contacting the principal investigator, Professor Milan Brazdil, M.D., PhD. A summary of the patient's description is provided in Supplementary Table 2. The feature description is provided in Supplementary Table 1. The computational library for iEEG feature extraction is publicly available at (https://gitlab.com/bbeer_group/development/epycom/epycom).
