# Supplementary material for: Leveraging interictal multimodal features and graph neural networks for automated planning of epilepsy surgery
Source: Brain Commun. 2025 Apr 16;7(3):fcaf140. doi: 10.1093/braincomms/fcaf140 (PMC12066826; doi:10.1093/braincomms/fcaf140)
Supplement: fcaf140_Supplementary_Data [file fcaf140_supplementary_data.zip › Supplementary_Table_1.docx]

Supplementary Table 1: Description of features extracted from the iEEG data.

|  | Feature |
| --- | --- |
| 1 | Signal power (intra-patient normalized between 0-1) |
| 2 | Spectral Centroid |
| 3 | Phase Amplitude Coupling  (phase: Delta 1-4 Hz, amplitude: Gamma) |
| 4 | Phase Amplitude Coupling  (phase: Delta 1-4 Hz, amplitude:  HFO-Ripples 80-250 Hz) |
| 5 | Phase Amplitude Coupling  (phase: Delta 1-4 Hz, amplitude:  HFO-Fast Ripples 200-600Hz) |
| 6 | Delta Beta Ratio (PIB_1-4_ / PIB_12-30_) |
| 7 | Delta power (intra-patient normalized between 0-1) |
| 8 | Power in band 0.1 - 1 Hz (relative to total signal power) |
| 9 | Power in band 1-4 Hz (relative to total signal power) |
| 10 | Power in band 4-8 Hz (relative to total signal power) |
| 11 | Power in band 8-12 Hz (relative to total signal power) |
| 12 | Power in band 12-30 Hz (relative to total signal power) |
| 13 | Power in band 30-80 Hz (relative to total signal power) |
| 14 | Power in band 45-65 Hz (relative to total signal power) |
| 15 | Power in band 80-250 Hz (relative to total signal power) |
| 16 | Power in band 200-600 Hz (relative to total signal power) |
| 17 | Epileptiform Discharges: absolute rate per minute (Janca et al, 2015) |
| 18 | Epileptiform Discharges: intra-patient relative rate per minute |
| 19 | Epileptiform Discharges propagation: sink index (Gunnarsdottir et al, 2022) |
| 20 | Epileptiform Discharges propagation: sink connectivity (Gunnarsdottir et al, 2022) |
| 21 | Epileptiform Discharges propagation: source index (Gunnarsdottir et al, 2022) |
| 22 | Epileptiform Discharges propagation: source influence (Gunnarsdottir et al, 2022) |
| 23 | Epileptiform Discharges propagation: network centrality (<https://networkx.org/documentation/stable/reference/algorithms/centrality.html#module-networkx.algorithms.centrality>) |
| 24 | Epileptiform Discharges propagation: network hub score (HITS algorithm, <https://networkx.org/documentation/stable/reference/algorithms/generated/networkx.algorithms.link_analysis.hits_alg.hits.html>) |
| 25 | Epileptiform Discharges propagation: network authority score (HITS algorithm) |
| 26 | Probability of being in white matter (based on MNI atlas) |
| 27 | Probability of being in gray matter (based on MNI atlas) |
| 28 | Probability of being in CSF (based on MNI atlas) |
| 29 | MNI position X |
| 30 | MNI position Y |
| 31 | MNI position Z |

**References**

Janca R, Jezdik P, Cmejla R, et al. Detection of interictal epileptiform discharges using signal envelope distribution modelling: application to epileptic and non-epileptic intracranial recordings. Brain Topogr. 2015;28(1):172-183. doi:10.1007/s10548-014-0379-1

Gunnarsdottir KM, Li A, Smith RJ, et al. Source-sink connectivity: a novel interictal EEG marker for seizure localization. Brain. 2022;145(11):3901-3915. doi:10.1093/brain/awac300
